# Pyrosequencing of 16S rRNA gene amplicons to study the microbiota in the gastrointestinal tract of carp (*Cyprinus carpio *L.)

**DOI:** 10.1186/2191-0855-1-41

**Published:** 2011-11-18

**Authors:** Maartje AHJ van Kessel, Bas E Dutilh, Kornelia Neveling, Michael P Kwint, Joris A Veltman, Gert Flik, Mike SM Jetten, Peter HM Klaren, Huub JM Op den Camp

**Affiliations:** 1 Department of Microbiology, IWWR, Radboud University Nijmegen, Heyendaalseweg 135, NL-6525 AJ Nijmegen, the Netherlands; 2 Department of Animal Physiology, IWWR, Radboud University Nijmegen, Heyendaalseweg 135, NL-6525 AJ Nijmegen, the Netherlands; 3 Center for Molecular and Biomolecular Informatics, Nijmegen Center for Molecular Life Sciences, Radboud University Nijmegen Medical Center, Geert Grooteplein 28, NL-6525 GA Nijmegen, the Netherlands; 4 Departments of Computer Science and Biology, San Diego State University, 5500 Campanile Drive, San Diego CA 92182, USA; 5 Department of Human Genetics, Radboud University Nijmegen Medical Centre, Geert Grooteplein 10, NL-6525 GA Nijmegen, the Netherlands

**Keywords:** intestinal tract, biodiversity, carp, aquaculture, pyrosequencing, 16S rRNA

## Abstract

The microbes in the gastrointestinal (GI) tract are of high importance for the health of the host. In this study, Roche 454 pyrosequencing was applied to a pooled set of different 16S rRNA gene amplicons obtained from GI content of common carp (*Cyprinus carpio*) to make an inventory of the diversity of the microbiota in the GI tract. Compared to other studies, our culture-independent investigation reveals an impressive diversity of the microbial flora of the carp GI tract. The major group of obtained sequences belonged to the phylum *Fusobacteria*. *Bacteroidetes*, *Planctomycetes *and *Gammaproteobacteria *were other well represented groups of micro-organisms. *Verrucomicrobiae*, *Clostridia *and *Bacilli *(the latter two belonging to the phylum *Firmicutes*) had fewer representatives among the analyzed sequences. Many of these bacteria might be of high physiological relevance for carp as these groups have been implicated in vitamin production, nitrogen cycling and (cellulose) fermentation.

## Introduction

The intestine is a multifunctional organ system involved in the digestion and absorption of food, electrolyte balance, endocrine regulation of food metabolism and immunity against pathogens (Ringo et al. 2003). The gastrointestinal (GI) tract is inhabited by many different micro-organisms. As in mammals, this dynamic population of micro-organisms is of key importance for the health of the piscine host (Ringo et al. 2003; Rawls et al. 2004). The gut is also a potential route for pathogens to invade and infect their host. The micro-organisms in the GI tract are involved in the protection against these pathogens by the production of inhibitory compounds and competition for nutrients and space. As in mammals, the intestinal microbiota of fish can influence the expression of genes involved in epithelial proliferation, nutrient metabolism and innate immunity (Rawls et al. 2004). Due to their importance in animal health, the investigation of the intestinal microbiota of fish is highly relevant for aquaculture practice. We investigated the diversity of the microbiota in common carp (*Cyprinus carpio*), one of the most cultivated freshwater fish species worldwide (FAO, 2011).

The morphology of the GI tract of fishes varies greatly among species. Common carp belong to the family of *Cyprinidae*, which are herbivorous, stomachless fish. These fish lack pyloric caeca, the finger-like blind sacs in the proximal intestine that increase the absorptive surface of the intestines in many fish (Ringo et al. 2003; Buddington and Diamond 1987). The composition of the gut microbiota of common carp has previously been investigated using culture-dependent methods (Sugita et al. 1990; Namba et al. 2007; Tsuchiya et al. 2008). Most bacterial species found in these studies were aerobes and facultative anaerobes. Two studies demonstrated a high abundance of *Aeromonas *species (Namba et al. 2007; Sugita et al. 1990). Other bacteria isolated were *Enterobacteriaceae *(Sugita et al. 1990; Namba et al. 2007), *Pseudomonas *(Sugita et al. 1990; Namba et al. 2007), *Bacteriodetes *(Sugita et al. 1990; Tsuchiya et al. 2008)*, Plesiomonas *(Sugita et al. 1990), *Moraxella *(Sugita et al. 1990; Namba et al. 2007), *Acinetobacter *(Sugita et al. 1990; Namba et al. 2007), *Flavobacterium *(Sugita et al. 1990), *Staphylococcus *(Sugita et al. 1990), *Micrococcus *(Sugita et al. 1990; Namba et al. 2007), *Streptococcus *(Sugita et al. 1990), *Bacillus *(Sugita et al. 1990), *Clostridium *(Sugita et al. 1990), *Vibrio *(Namba et al. 2007) and *Cetobacterium *(Tsuchiya et al. 2008). However, these studies only reveal the microbes that can be cultured and these most likely do not reflect the complete microbial composition of the carp gut since studies on mammals have shown that most members of the microbiota in the GI tract cannot be cultured when removed from the gut (Suau et al. 1999; Moya et al. 2008). The use of culture-independent studies such as molecular screening of the 16S rRNA gene may be a more reliable method to estimate microbial diversity in the GI tract of fish (Wu et al. 2010). Next generation sequencing is a powerful technique to investigate the composition of complex microbial communities in different environments (Hong et al. 2010; Qin et al. 2010; Vahjen et al. 2010;Moya et al. 2008; Kip et al. 2011; Roeselers et al. 2011). The combination of 16S rRNA gene amplification using multiple primer sets and the subsequent sequencing of the PCR products by Roche 454 pyrosequencing should therefore be a powerful method to assess the diversity of the microbiota in the GI tract of common carp. Obtained 16S rRNA gene sequences were used to classify the different microorganisms present in the fish gut and here we will also discuss the possible functions of these bacteria in the carp gut.

## Materials and methods

### Fish and system configuration

Common carp (*Cyprinus carpio *L.) were kept in 140 L tanks in a closed recirculating aquaculture system with a total volume of 3000 L at the Radboud University Nijmegen (The Netherlands). Fish were fed commercial food (Trouvit, at a daily ration of 1% estimated body weight), containing 45% protein. Water quality of the system was maintained by a biofilter and a weekly water replacement of 10% of the total volume. Ten fish (male and female) weighing 60 to 158 gram were used. All experimental procedures were performed with permission of the local ethical review committee (Radboud University Nijmegen).

### DNA extraction, PCR amplification and sequence analysis

Ten fish were euthanized using 0.1% ethyl-m-aminobenzoate methane sulfonate salt (MS-222, MP Biomedicals, Illkirch, France, pH adjusted to 7) followed by decapitation. The body surface of the fish was washed with 70% ethanol and the GI tract was removed aseptically. The whole content of the GI tract was removed by carefully flushing with PBS and DNA was extracted from this material using a cetyltrimethylammoniumbromide (CTAB)-based extraction method (Zhou et al. 1996). Briefly, samples were mixed with CTAB-extraction buffer (100 mM Tris-HCl (pH 8.0), 100 mM EDTA, 100 mM sodium phosphate (pH 8.0), 1.5 M NaCl, 1% CTAB, 675 μl per 250 mg sample) and protease K (10 mg/ml) and incubated for 30 min at 37°C. After protease treatment 10% SDS was added, followed by incubation at 65°C for 2 h. DNA was recovered by phenol/chloroform extraction and ethanol precipitation and the resulting DNA pellet was resuspended in 1 ml ultrapure water. Before additional purification, DNA was treated with RNAse. The DNA thus obtained was purified using Sephadex beads (Amersham Bioscience, USA) according to the manufacturer's protocol and its integrity was checked on agarose gel. DNA concentrations were estimated spectophotometrically using NanoDrop^® ^technology (Thermoscientific, USA).

### Retrieval of 16S RNA gene sequences

Obtained DNA (20 ng) was used for amplification in 20 μl reactions using Phusion Flash enzymes (Finnzymes, Finland). In order to target as many bacterial taxa as possible, the Pla46 F primer was combined with EubI R, EubII R or EubIII R and for the 616 F primer the same set of reverse primers was used (Table 1). This resulted in 6 different combinations. All reactions were done for individual fish separately. PCR reactions were started by an initial denaturation at 98°C for 1 min followed by 35 amplification cycles (98°C for 6 s, 10 s at annealing temperature, 72°C for 20 s) and a final extension step for 1 min at 72°C. PCR products were examined for size and yield using agarose gel in TAE buffer (20 mM Tris-HCl, 10 mM sodium acetate, 0.5 mM Na_2_EDTA, pH 8.0). After successful amplification, obtained products of different reactions were pooled and 9.2 μg PCR product was used for pyrosequencing using the Roche 454 GS FLX Titanium sequencer (Roche, Switzerland). A problem with 454 pyrosequencing is 'blinding' of the camera due to flashing caused by incorporation of the same nucleotide in many spots, which can occur when many similar DNA templates are sequenced (Kip et al. 2011). This was circumvented by mixing 16S rRNA gene products in a 1:1 ratio with *pmoA *PCR products (targeting a subunit of the particulate methane monooxygenase) from a non-related experiment (Kip et al. 2011).

**Table 1:** Primer specifications

Primer	Target	Sequence (5'-3')	Reference
**Pla46 F**	*Planctomycetales*	GGATTAGGCATGCAAGTC	Neef et al. 1998
**616 F**	Most bacteria	AGAGTTTGATYMTGGCTCAG	Juretschko et al. 1998
**EubI R**	Most bacteria	GCTGCCTCCCGTAGGAGT	Amann et al. 1990
**EubII R**	*Planctomycetales*	GCAGCCACCCGTAGGTGT	Daims et al. 1999
**EubIII R**	*Verrucomicrobiales*	GCTGCCACCCGTAGGTGT	Daims et al. 1999

### Phylogenetic analysis

A Megablast search (using default parameters) of all sequenced reads larger than 100 nt against the Silva SSURef database (version 102) was done to extract all 17,892 16S rRNA gene sequences (average length 314 nt). The taxonomic annotations available in the Silva SSURef database were used to classify the sequenced reads. Each read was assigned to the taxonomic clade of its highest scoring Megablast hit, when a sequence was assigned to more than one clade, its vote was divided equally. Furthermore, obtained sequences were processed using the Classifier tool (Wang et al. 2007) of the RDP pyrosequencing pipeline http://pyro.cme.msu.edu/. The confidence threshold used was 50%. The sequence reads are available at the MG-Rast Metagenome analysis server http://metagenomics.anl.gov/ under Project ID 4449604.3 and from the Sequence Read Archive (SRA) at http://www.ebi.ac.uk/ena/data/view/ under accession number ERP000995.

## Results

The use of next generation sequencing technologies for sequencing of a mixture of 16S rRNA amplicons amplified with primer sets targeting as many phyla as possible will give a much broader taxonomic overview compared to the use 16S rRNA hypervariable regions (Kysela et al. 2005). To avoid missing a certain group of bacteria, different primer sets (Table 1) were used targeting as much species as possible. Obtained amplicons from all different reactions were mixed and sequenced using Roche 454 titanium technology and this revealed a high microbial diversity in the GI tract of common carp (*Cyprinus carpio*). It should be noted that the use of multiple primer sets biases the number of sequences belonging to the identified taxa. The number of obtained sequences belonging to a specific group may not be representative for their abundances *in vivo*; therefore no quantitative statements could be made.

**Figure 1:**
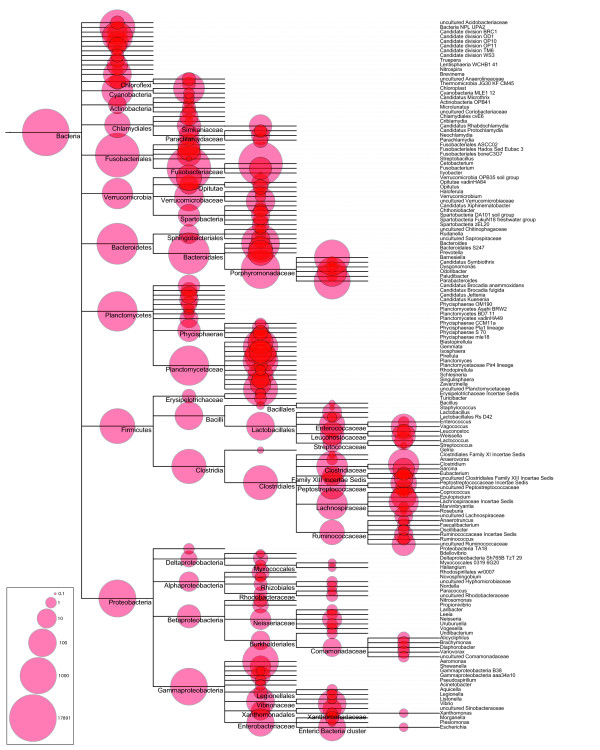
**Phylogenetic diversity of 16S rRNA sequences retrieved from the GI tract content of common carp**. Clasification the 17,641 reads was performed using the taxonomic annotations available in the Silfva SSURef database. The number of sequences (10log-transformed) belonging to each clade is indicated by the red circles.

Figure 1 displays the taxonomic classification derived from mapping the pyrosequencing reads to the Silva SSURef database, which classified 17,641 reads (99%). Similar results were obtained when the RDP database pyrosequencing pipeline was used, which classified 16,768 reads (94%, Additional file 1). Almost half of the obtained sequences, i.e. 46%, found belonged to the *Fusobacteria *(Additional file 2). Other well represented groups within the retrieved sequences were the *Bacteroidetes *(21%), *Planctomycetes *(12%), and *Gammaproteobacteria *(7%); less retrieved sequences belonged to the *Clostridia *(3%), *Verrucomicrobiae *(1%), and *Bacilli *(1%). Furthermore, a few sequences (< 1%) were identified as *Opitutae*, *Chlamydiae*. *Verrucomicrobiae *subdivision 3, *Betaproteobacteria *and *Nitrospira *were also detected (Additional file 2). 77 sequences were classified as cyanobacteria-like, probably these are chloroplast sequences that originate from the plant components of the food (Additional file 2). Interestingly, most of the retrieved sequences belong to bacterial taxa that are known to be involved in vitamin production and food digestion (Table 2).

**Table 2:** Niche and possible function of the bacterial classes present within the 16S rRNA amplicons obtained from the GI tract of common carp.

Class/Subclass	Phylum	Metabolism	Niche and function	Reference
***Aeromodales***	*Proteobacteria*	Facultative anaerobes	Well-known pathogen in fish, known member of the endogenous flora of freshwater fish, fermentation of organic compounds, cellulose activity, antibacterial activity	Lee et al. 2009; Huber et al. 2004; Namba et al. 2007; Wu et al. 2010; Jiang et al. 2011; Sugita et al. 1995; Sugita et al. 1997
***Bacilli***	*Firmicutes*	Aerobic heterotrophs	Bacilli, especially lactobacilli, are known members of the microbial flora of the fish gut, able to ferment various carbon hydrates, pathogens	Ringo and Gatesoupe 1998
***Bacterioidaceae***	*Bacteriodetes*	Obligate anaerobes	Polysaccharide (especially from plants) degradation, known member of the intestinal microbiota of various organisms	Van der Meulen et al. 2006; Flint et al. 2008
***Cetobacterium***	*Fusobacteria*	Obligate anaerobes	Known member of the endogenous flora of fish intestines, vitamin B_12 _production	Sugita et al. 1991; Wu et al. 2010; Tsuchiya et al. 2008
***Clostridia***	*Firmicutes*	Obligate anaerobes	Known member of the endogenous flora of intestines of various organisms including fish, polysaccharide degradation, pathogen, antibacterial activity	Flint et al. 2008; Wu et al. 2010; Sugita et al. 1990; Sugita et al. 1997
***Enterobacteriales***	*Proteobacteria*	Facultative anaerobes	Sugar fermentation, pathogen, known member of the intestinal microbiota of fish (including carp)	Wu et al. 2010; Sugita et al. 1990
***Gemmata***	*Planctomycetes*	Aerobic heterotrophs	Abundant in freshwater ecosystems	Wang et al. 2002
***Isosphaera***	*Planctomycetes*	Aerobic hetetotrophs	Common in aquatic environments	Wang et al. 2002
***Marinilabiaceae***	*Bacteriodetes*	Facultative anaerobic chemo-organotrophs	Sugar/starch fermentation, members of this family can decompose plant polymers and some have low cellulose activity	Denger et al. 2002; Detkova et al. 2009
***Pirellula***	*Planctomycetes*	Aerobic heterotrophs	Carbohydrate fermentation, present in aquatic environments, present in guts of some animals and associated to sponges	Fuerst et al. 1997; Pimental-Elardo 2003
***Planctomyces***	*Planctomycetes*	Aerobic heterotrophs, anaerobic chemoautotrophs	Known member of the intestinal microbiota of various organisms including fish	Ley et al. 2008; Rawls et al. 2006
***Porphyromonadaceae***	*Bacteriodetes*	Obligate anaerobes	Pathogen, major members of the human gut microbiota, present in fish intestines, glucose fermentation	Mulder et al. 2009; Wu et al. 2010; Li et al. 2009
***Schlesneria***	*Planctomycetes*	Facultative aerobic chemo-organotrophs	Present in wetlands, degradation of biopolymers	Kulichevskaya et al. 2007
***Sphingobacteria***	*Bacteriodetes*	Obligate anaerobes	Endosymbiont in insects, plant polysaccharide degradation	Zhou et al. 2009
***Verrucomicrobiae***	*Verrucomicrobia*	Aerobes, facultative anaerobes	Fermentation, known members of the fish microbiota	Schlesner et al. 2006; Rawls et al. 2006
***Vibrio***	*Proteobacteria*	Facultative anaerobes	Fermentation, pathogen, obligate endosymbionts, known to be present in fish intestines	Wu et al. 2010; Thompson et al. 2004
***Zavarzinella***	*Planctomycetes*	Aerobic heterotrophs	Acidic wetlands, newly identified genus related to *Gemmata*	Kulichevskaya et al. 2009

## Discussion

Almost all Fusobacterial 16S rRNA sequences, 8081 out of 8085, from the carp GI tract belonged to the genus *Cetobacterium*. *Cetobacteria *were not observed in most culture-dependent studies done on the GI tract microbiota of common carp (Sugita et al. 1990; Namba et al. 2007), only Tsuchiya et al. (2008) described the isolation and characterization of *Cetobacterium somerae *from the GI tract of five different fresh water fish, including carp. *Cetobacterium *was also shown to be present in the gut of zebrafish (Rawls et al. 2006), a cyprinid species closely related to common carp. Furthermore, *Cetobacterium *isolated from human faeces performed fermentation of peptides and carbohydrates (Finegold et al. 2003). It has also been shown that *Cetobacterium *can produce vitamin B12 (Tsuchiya et al. 2008). This can wel explain why carp do not have a dietary vitamin B12 requirement (Sugita et al. 1991). The combination of a fermentative metabolism together with vitamin production may explain the relevance of *Cetobacterium *sp. in the GI tract of carp.

Another well represented group within the obtained sequences were the *Bacteroidetes *(22% of obtained sequences), a phylum known for a fermentative metabolism and degradation of oligosaccharides derived from plant material (Van der Meulen et al. 2006). The *Bacteroidetes *sequences found could be divided into 4 major groups (Additional file 1): *Marinilabiaceae *(or *Cytophaga*, 13%), *Porphyromonadaceae *(39%), *Bacteroidaceae *(15%) and *Bacteroidales_incertae_sedis *(33%). All *Marinilabiaceae *sequences belonged to the same group: the *Anaerophaga*. This relatively newly discovered group of bacteria includes strictly anaerobic, chemo-organotrophic, fermentative bacteria (Denger et al. 2002). These bacteria may play an important role in the fermentation of food in the GI tract of herbivorous carp since anaerobic fermentation is generally an important step in the digestion of plant material. *Porphyromonadaceae *are present in the GI tract of several organisms including human and pigs (Mulder et al. 2009). These bacteria can be pathogens but in this niche they are most probably involved in fermentation. By using labelled glucose, it has been shown that these bacteria are involved in saccharide fermentation (Li et al. 2009). Also the *Sphingobacteria *present could also be involved in oligosaccharide degradation since *Sphingobacterium *sp. TN19, an endosymbiont in insects, contains a xylanase encoding gene (Zhou et al. 2009). Xylanases are involved in the breakdown of xylan, a polysaccharide found in plant material. The presence of fermenting microorganisms is not suprising, since it has been shown that the GI microbiota of carp is able to ferment different oligosacharides (Kihara and Sakata 2002).

The obtained *Planctomycete *sequences (13% of classified sequences) could be divided into 9 groups (Additional file 1); *Gemmata*, *Pirellula*, *Schlesneria *and *Zavarzinella *were the most abundantly found groups. *Gemmata *and *Pirellula *are aerobic chemo-heterotrophs, *Schlesneria *are chemo-organotrophic facultative aerobes and *Zavarzinella *are aerobic heterotrophs. The presence of *Planctomycetes *has been shown before in gut microbiota of fish and other organisms (Ley et al. 2008; Rawls et al. 2006). The exact function of these bacteria in the GI tract is not clear, possibly these bacteria live from products of the metabolism of other bacteria. However, the relatively high abundance of *Planctomycetes *in close association with other organisms such as kelp, marine sponges and prawn (Bengtsson and Ovreas 2010; Pimental-Elardo 2003; Fuerst et al. 1997; Lahav et al. 2009) suggests a more important role. Possibly, these bacteria are involved in the metabolism of complex compounds. In a recent study, in which the close association of *Planctomycetes *with the brown seeweed kelp (*Laminaria hyperborea*) was investigated, it was hypothesized that these bacteria are degraders of sulfated polysacharides produced by kelp (Bengtsson and Ovreas 2010). The organisms found in the biofilm at the plant's surface were mainly members of the lineage *Pirellulae *(which includes *Pirellula, Rhodopirellula *and *Blastopirellula*). The genome sequence of *Rhodopirellula baltica *SH1 revealed many genes involved in the breakdown of sulfated polysaccharides (Glockner et al. 2003). Possibly, the heterotrophic *Planctomycetes *found in carp gut confer a similar ability of polysaccharide breakdown to the host. Furthermore, a separate lineage within the *Planctomycetes*, the anammox bacteria, were present in the carp gut (Figure 1). These anaerobic bacteria, described before in fish gut (Lahav et al. 2009), are involved in nitrogen cycling. Together with the *Nitrosomonas *and *Nitrospira *species (also present within the obtained sequences, Figure 1), ammonium can be converted into dinitrogen gas. The removal of nitrogenous compounds from aquaculture systems is one of the most important challenges in aquaculture. The presence of nitrogen cycling bacteria in fishes could offer new *in situ *solutions for the removal of nitrogen from aquaculture systems.

The *Gammaproteobacteria *sequences found could be classified as bacteria that are known members of the GI microbiota of many organisms including fish (Wu et al. 2010; Lee et al. 2009). Most *Gammaproteobacteria *(Additional file 1) found in carp belonged to the *Aeromonas *group. Members of the genus *Aeromonas *are mainly distributed in freshwater and sewage, often in association with aquatic animals (Cahill 1990; Sugita et al. 1995). They can cause a diverse spectrum of diseases in both warm- and cold-blooded animals but they also appear to be aquatic envrionments including in fish intestines (Sugita et al. 1995). Other abundantly present members among the Gammaproteobacterial sequences were the genera *Enterobacterium *and *Vibrio*. *Enterobacterium *spp. are widespread in GI tracts of various organisms (Wu et al. 2010), whereas *Vibrio *sp. are commonly found in aquaeous environments, aquaculture systems and in association with eukaryotes (Wu et al. 2010; Thompson et al. 2004). This phylum also contains *Plesiomonas *and *Acinetobacter *species that have been found in carp before (Sugita et al. 1991; Cahill 1990). Furthermore, the presence of high number *Proteobacteria *has also been shown for zebrafish, which is closely related to carp (Rawls et al. 2006). Also in other fish belonging to the *Cyprinidae *members of the *Gammaproteobacteria *(*Enterobacter *and *Citrobacter *species) were found (Ray et al. 2010). *Enterobacter *and *Citrobacter *species isolated from the GI tract of Indian carp (*Cyprinidae*) were shown to produce amylase, cellulase and protease (Ray et al. 2010), which indicates that these bacteria can be actively involved in the digestion of food in carp guts.

Another abundant phylum within our amplicon sequences were the *Verrucomicrobiae *(including subdivision 3 and 4 (*Optitiae*)). *Verrucomicrobiae *species are most commonly found in aquatic environments but are also known members of the gut microbiota in different organisms including seacucumbers (*Echinodermata*), termites and humans (Wagner and Horn 2006). These bacteria seem to be well adapted to live with eukaryotes, since the genome of some verrucomicrobial species contain a protein secretion system which mediates interactions between eukaryotic and bacterial cells (Wagner and Horn 2006). *Verrucomicrobiae *usually have an aerobic or obligate anaerobic fermentative metabolism (Schlesner et al. 2006) and could also play a role in the metabolism of plant beta glycans in carp GI tract. Indeed, *Pedosphaera parvula *Ellin514 (*Verrucomicrobia *subdivision 3) contains a cellulase in its genome (Kant et al. 2011). Ruminants and postgastric fermenters depend on bacteria containing this gene for the fermentation of plant material in which cellulose is converted to β-glucose. Various fish species do have a cellulase activity in their guts (Saha and Ray 1998; Saha et al. 2006; Ray et al. 2010) which decreases after antibiotic treatments (Saha and Ray 1998), indicating that the GI microbiota is responsible for this activity.

*Clostridia *and *Bacilli*, both present in the microbiota of the sampled fish (Figure 1), are members of the phylum *Firmicutes*. Representative genera of this phylum, including *Clostridium*, *Bacillus*, *Streptococcus *and *Staphylococcus *spp., have been shown in the microbiota of fish before (Navarrete et al. 2009; Rawls et al. 2006; Ray et al. 2010; Sugita et al. 1990). Gut isolates belonging to the *Firmicutes *fermented various carbon sources (Ray et al. 2010), again implicating a role in the utilization of plant materials.

To our knowledge, this is the first detailed analysis of the microbiota of common carp by high throughput sequencing. Our culture independent investigation of the microbial flora of the GI tract gives a more reliable and more complete characterization of the diversity of compared to other studies. Furthermore, great similarities between the microbiota in carp and zebrafish (a closely related fish species) were shown (Roeselers et al. 2011). The GI microbiota is important for the health of the animal and therefore this study could be relevant for aquaculture. Furthermore, the presence of different nitrogen cycling bacteria in the GI tract of fish could offer new possibilities in the removal of nitrogen compounds in aquaculture. The microbiota of the GI tract plays an important role in the digestion and chemical processing of the food as exemplified by the large number of bacteria involved in vitamin production and fermentation of saccharides and beta-glycans (cellulose, hemicellulose) (Table 2). The presence of many different types of bacteria in the herbivorous carp could be predicted since it has been shown that eukaryotes with an herbivorous diet have a higher microbial diversity (Ley et al. 2008). However, the carp in our study were fed commercially available food with high protein and low plant content. According to their GI microbiota, these fish are very well able to adapt to a more herbivorous diet and this is probably also the case for other cultured fish. Therefore it could be possible to lower the amount of fish meal, one of the major components of fish food, in the food for these fish. Furthermore, it shows that the gut microbes are probably important in the protection of the host against pathogens which should be taken into consideration in aquaculture where a lot of antibiotics are used (Cabello 2006). It is known that antibiotics have a negative effect on the microbial community in the gut of human (Dethlefsen et al. 2008) and this is possibly also the case for fish. The routinely use of antibiotics may be harmful for the animal. A better knowledge about the microbiota in fish guts is important; it can lead to a better health of cultured fish and therefore to a more efficient fish culture.

## Competing interests

The authors declare that they have no competing interests.

## Supplementary Material

Additional file 1 **Phylogenetic diversity of the bacterial 16S rRNA sequences. Supplemental Figure S1**. Click here for file

Additional file 2 **Details of the phylogenetic composition of the bacterial sequences. Supplemental Table S1**. Click here for file
