# Supplementary material for: Pyrosequencing of 16S rRNA gene amplicons to study the microbiota in the gastrointestinal tract of carp (Cyprinus carpio L.)
Source: AMB Express. 2011 Nov 18;1:41. doi: 10.1186/2191-0855-1-41 (PMC3226434; doi:10.1186/2191-0855-1-41)
Supplement: Additional file 1 — Phylogenetic diversity of the bacterial 16S rRNA sequences. Supplemental Figure S1. [file 2191-0855-1-41-S1.PDF]

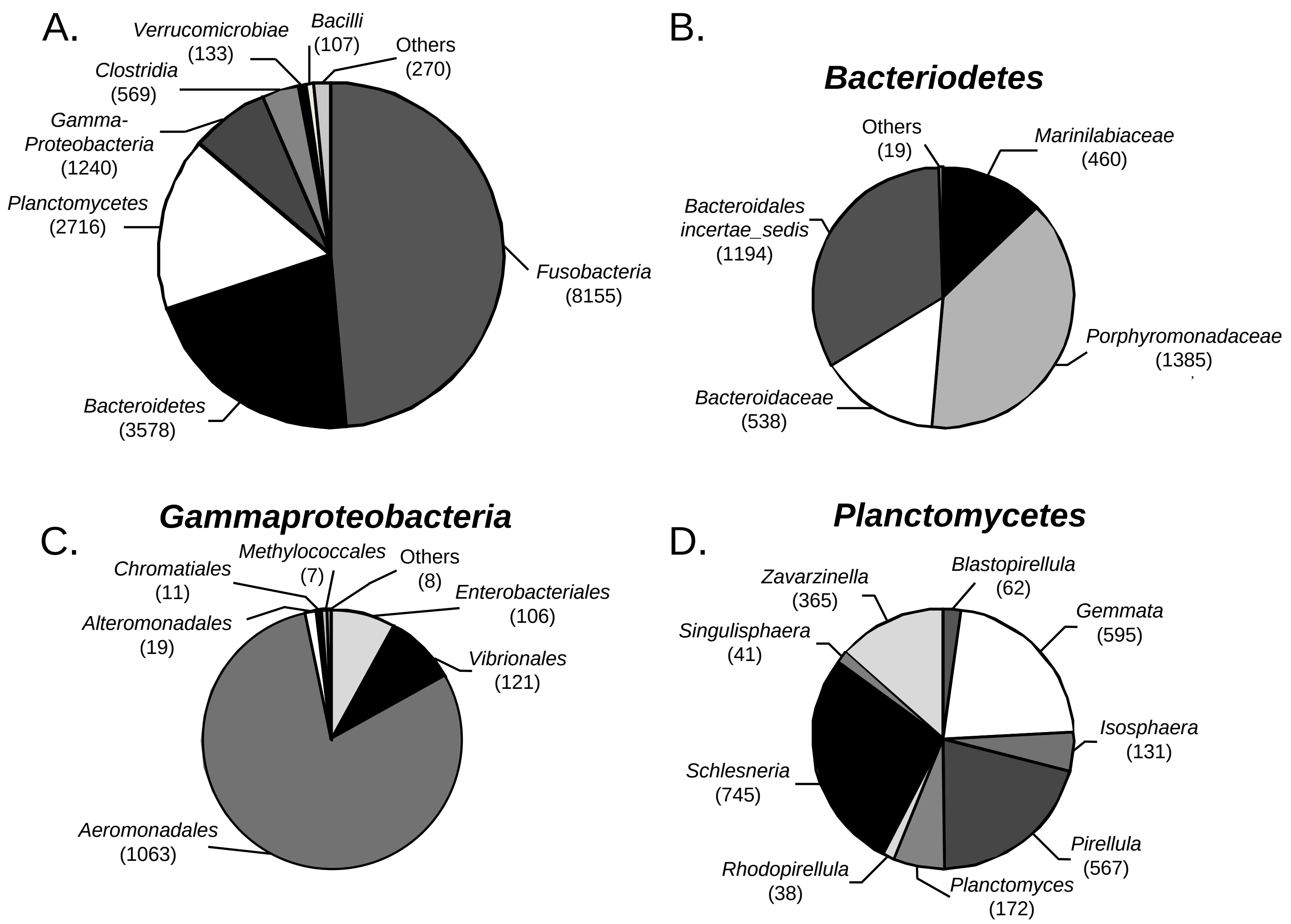

Supplemental Fig. S1. A. Phylogenetic diversity of the bacterial 16S rRNA sequences retrieved from the content of the GI tract of common carp (*Cyprinus carpio* L.), determined using the Classifier tool (Wamg et al. 2007) of the RDP pyrosequencing pipeline. Composition of the largest groups is shown in more detail (**B**, **C**, **D**). Number of sequences are given between brackets.
