# Supplementary material for: Pyrosequencing of 16S rRNA gene amplicons to study the microbiota in the gastrointestinal tract of carp (Cyprinus carpio L.)
Source: AMB Express. 2011 Nov 18;1:41. doi: 10.1186/2191-0855-1-41 (PMC3226434; doi:10.1186/2191-0855-1-41)
Supplement: Additional file 2 — Details of the phylogenetic composition of the bacterial sequences. Supplemental Table S1. [file 2191-0855-1-41-S2.PDF]

Supplemental Table S1. Details of the phylogenetic composition of the bacterial sequences from the content of the GI tract of common carp (*Cyprinus carpio*), determined by Megablast mapping against the Silva SSURef database (vs 102).

**Bacterial group**	**Number of Sequences**	**Percentage of sequences**
Actinobacteria	2	0.01
Bacteriodetes	3654	20.7
BRC1_genera_incertae_sedis	11	0.1
Chlamydiae	58	0.3
Chloroflexi	1	0.01
Chloroplast	77	0.4
Deinococcus-Thermus	3	0.02
Firmicutes (*Bacilli*)	107	0.6
Firmicutes (*Clostridia*)	562	3.2
Firmicutes (*Erysipelotrichi*)	1	0.01
Fusobacteria	8085	45.8
Nitrospira	1	0.01
OP11_genera_incertae_sedis	1	0.01
Planctomycetes	2181	12.4
Proteobacteria (*alpha*)	7	0.04
Proteobacteria (*beta*)	39	0.2
Proteobacteria (*delta*)	11	0.1
Proteobacteria (*gamma*)	1185	6.7
Proteobacteria (unclassified)	2	0.01
Spirochaetes	112	0.6
Division OD1	6	0.03
Verrucomicrobia	232	1.3
Unclassified	1305	7.4
